# Effects of the COVID-19 pandemic on the mental health of Japanese university students without depressive symptoms at university entrance

**DOI:** 10.1186/s13030-025-00329-9

**Published:** 2025-07-04

**Authors:** Yoshie Miyake, Koki Takagaki, Atsuo Yoshino, Yuri Okamoto

**Affiliations:** Health Service Center, Hiroshima University, 1-7-1, Kagamiyama, Higashi-Hiroshima, Hiroshima, 739-8514 Japan

**Keywords:** COVID-19 pandemic, Depressive symptom, Eating behavior, Stress coping, University students

## Abstract

**Background:**

The COVID-19 pandemic had a negative impact on the mental health of university students. However, despite the many downsides of experiencing the pandemic, some students also experienced positive consequences, such as reduced academic pressure and increased time to attend to wellness. The impact may vary from individual to individual. This study investigated the effects of the pandemic on the mental health of Japanese university students who did not have depressive symptoms before the pandemic.

**Methods:**

The data of 3,100 fourth-year university students who did not have depressive symptoms at either the clinical or subthreshold level, or disordered eating behaviors at university entrance were available for analysis: 1,335 were fourth-year students before the pandemic and 1,765 fourth-year students during the pandemic. Differences in depressive symptoms were examined with the Beck Depression Inventory II (BDI-II), eating behaviors with the Eating Attitudes Test-26 (EAT-26) and Bulimic Inventory Test, Edinburgh (BITE) and stress coping with Coping Inventory for Stressful Situations (CISS). Differences in the frequencies of clinical, subthreshold, and nondepressed depression status groups were compared for before and during the pandemic groups. Furthermore, we investigated the relation between the development of depression and stress coping styles during the pandemic.

**Results:**

The BDI-II and BITE scores of fourth-year students during the pandemic were significantly lower than those before the pandemic. Female fourth-year students during the pandemic scored significantly higher on the CISS-task-oriented scale than did fourth-year students before the pandemic. Changes in stress coping behavior were also associated with the development of depressive symptoms during the pandemic.

**Conclusion:**

Fourth-year university students who did not have depressive symptoms or disordered eating behaviors at university entrance experienced fewer depressive symptoms and fewer bulimic symptoms during the pandemic than did students before the pandemic. Additionally, our results suggest that task-oriented was associated with a decreased risk of depressive symptoms during the pandemic, especially for female students.

## Introduction

During the coronavirus disease 2019 (COVID-19) pandemic, people were exposed to high-stress and previous studies revealed that the prevalence of mental health problems increased over time in various populations [1]. Younger individuals seemed to be more severely affected by the pandemic than older persons [2]. After the start of the pandemic, university students faced restrictions regarding on-campus classes and club activities. They were exposed to high levels of COVID-19-related stress, and mental health effects have been reported [3–6]. Several studies have reported that the incidence of depressive symptoms in university students during the pandemic was greater than that before the pandemic [3, 7–9]. Stressful situations are associated with various behavioral responses including disordered eating behaviors [10]; a study in Japan suggested that the pandemic might have increased the prevalence of eating disorders (EDs) [11]. Restrictions due to the pandemic increased the risk of developing or worsening EDs due to disrupted eating and exercise habits, social isolation, lack of support, and limited access to healthcare [12, 13]. Previous studies reported that the onset of EDs increases from adolescence through the university period [14, 15]. To prevent EDs, investigating the effects of the pandemic on eating behaviors of university students is necessary.

A cohort study reported that almost one in five young adults felt better during than before the pandemic [16]. A recent study suggested that youth may benefit from more time to attend to health and wellness, potentially resulting in increased sleep and exercise [17]. Among university students, some students felt that the change in their environment during the pandemic had some good aspects. Staying at home and reduced academic pressures provided them with more time for relaxation and hobbies. A study of university students in Japan reported that there was no evidence that states of emergency exacerbated depressive states [18]. Despite its many negative consequences, the pandemic may have given some young people the opportunity to take stock of their lives and to improve their long-term well-being [16]. The impact may vary from individual to individual.

According to previous studies, the pandemic had a positive effect on the experiences of some university students, and there is some information available on the coping mechanisms that they benefited from [19]. Coping mechanisms are important for surviving in a pandemic situation [20, 21]. Several studies have suggested that the use of adaptive stress coping styles, such as cognitive and behavioral efforts to manage stressful conditions, can reduce the occurrence of stress‑related diseases and increase general health [22], and nonadaptive stress coping styles, such as emotional responses to problems and unhealthy behaviors, are risk factors for depression and EDs [23, 24]. The pandemic may have had not only a negative impact but also a positive impact on the depressive symptoms and eating behaviors of university students, especially those who tend to engage in adaptive stress coping. In high-stress situations such as the pandemic, there is a need to identify individuals and populations at greater risk of psychological distress and to offer targeted mental health care [25, 26].

The purpose of this study was to investigate the effects of the COVID-19 pandemic on depressive symptoms, eating behaviors, and stress coping behaviors among Japanese university students who did not have depressive symptoms, at the clinical or subthreshold level, or disordered eating behaviors at the time of university entrance. Both clinical and subthreshold depression are characterized by depressed mood and high psychiatric comorbidity [27]. The effects of the pandemic varied widely within and across groups [25]. However, only a few studies have investigated the effects of the pandemic on the mental health of students who did not have depressive symptoms before the pandemic. We compared the depressive symptoms, eating behaviors, and stress coping behaviors of students before and during the pandemic. We hypothesized that the pandemic would not worsen the depressive symptoms or disordered eating behaviors of fourth-year students who did not have those symptoms at the time of university entrance.

## Methods

### Participants

The participants were fourth-year students attending Hiroshima University from 2017–2019 (Time 1: before the pandemic) and in the 2021–2022 school year (Time 2: during the pandemic). Because the start of the new academic year coincided with the emergence of the pandemic and the schedule for health checkups was changed, the data of fourth-year students in 2020 were not included. The inclusion criteria were age 18–19 years at the time of university entrance, completing questionnaires at both university entrance and during the fourth year, not having depressive symptoms, including at the clinical or subthreshold level, and not having disordered eating behavior at university entrance.

### Procedures

The data was collected at two health checkup time points: university entrance and the first quarter of the fourth year. The questionnaires were administered as part of an annual checkup. Based on previous studies, the students were divided into two categories based on depressive symptoms determined by the Beck Depression Inventory II (BDI-II) [28] scores: students with depressive symptoms (clinical and subthreshold groups: BDI-II scores ≥ 10) and students without depressive symptoms (nondepressed group: BDI-II scores < 10) at university entrance [27, 29]. Screening for disordered eating behavior was by Eating Attitudes Test-26 (EAT-26) [30] and the Bulimic Inventory Test, Edinburgh (BITE) [31]. A score of 20 or more points on the EAT-26 and BITE indicates the presence of severely disordered eating behavior. Therefore, the data of students classified into the nondepressed group according to the BDI-II score and who scored 19 or less on the EAT-26 and BITE at university entrance were available for analysis as the non-depressed group.

To investigate the pandemic effects on the mental condition of fourth-year students, the BDI-II, EAT-26, BITE, and Coping Inventory for Stressful Situations (CISS) [32] scores before (Time 1) and during (Time 2) the pandemic were compared. Moreover, we examined whether differences in stress coping ability existed between students whoes condition deteriorated from nondepressed to subthreshold or clinical depression and those who remained nondepressed during the pandemic.

The study protocol was reviewed and approved by the Ethics Committee of the Hiroshima University School of Medicine, Japan (approval number E-2019–1767) and conducted ethically in accordance with the Declaration of Helsinki. Although the questionnaires were performed as part of an annual checkup, informed consent was obtained in the form of opt-out.

### Measures

#### Beck Depression Inventory II (BDI-II)

The original BDI-ll [28] consists of 21 self-reported items rated on a 4-point scale and is used to measure depressive symptoms; the cutoff point for clinical depression is a score of 18. A cutoff score of 18 yielded a sensitivity of 94% and a specificity of 92%, and a cutoff score of 10 yielded a sensitivity of 100% and a specificity of 70% [29]. Our clinical group included students with a BDI-II score of 18 or more, the subthreshold group those with scores ranging from 10 to 17, and the nondepressed group those with scores of 9 or less. The Cronbach's alpha coefficient was 0.87 [33]. The reliability and validity of the Japanese version of the BDI-II have been demonstrated [34].

#### Eating Attitudes Test-26 (EAT-26)

The EAT-26 is a reliable and valid 26-item self-report questionnaire that assesses eating attitudes [30]. Answers are provided on a 6-point scale ranging from “not at all” to “extremely”. The cutoff score is 20 points; scores greater than 20 indicate a high possibility of an eating disorder. Mann et al. [35] reported that a threshold of 20 yielded a sensitivity of 88%, and a specificity of 96%. The Cronbach’s alpha coefficients ranged from 0.85–0.94 [36]. The reliability and validity of the Japanese version of the EAT-26 have been demonstrated [37, 38].

#### Bulimic Inventory Test, Edinburgh (BITE)

The BITE is a self-reported measure of bulimic symptoms that consists of a symptom evaluation scale (30 items) and a severity scale (6 items) [31]. The symptom evaluation scale is scored as yes or no, and it has a minimum score of 0 and a maximum score of 30. The cutoff score is 20 points; a symptom subscale score greater than 20 indicates the presence of binge-eating behavior and a high possibility of bulimia nervosa. The Cronbach’s alpha coefficient was 0.96. The reliability and validity of the Japanese version of the BITE have been demonstrated [39].

#### Coping Inventory for Stressful Situations (CISS)

The CISS is a 48-item self-report measure scored on a 5-point scale that consists of three subscales to evaluate coping behaviors: task-oriented (CISS-T) (solving a problem, cognitive restructuring of a problem, or attempts to alter a situation), emotion-oriented (CISS-E) (emotional responses to a problem), and avoidance-oriented (CISS-A) (seeking distractions) coping [32, 40]. The reliability and validity of the Japanese version of the CISS have been demonstrated [41]. The Cronbach’s alpha coefficients range from 0.75–0.89 [41].

### Data analysis

SPSS version-28 (IBM Corporation, Armonk, NY) was used for the statistical analyses. Participant characteristics were averaged. To investigate the impact by sex, analyses were conducted separately for male and female students. The two-way repeated-measures analysis of variances (ANOVAs) was used to compare the scores by grade (first-year and fourth-year) and time (Time 1 and Time 2). Chi-square and residual analyses was used to compare the frequencies of depressive symptoms among the three groups. To investigate the relation between the development of depressive symptoms and coping style, we conducted two-way repeated-measures ANOVAs to compare the CISS scores by grade and group of depressive symptoms during the pandemic. The statistical significance level was set to *p* < 0.05.

## Results

### Participants

**Fig. 1:**
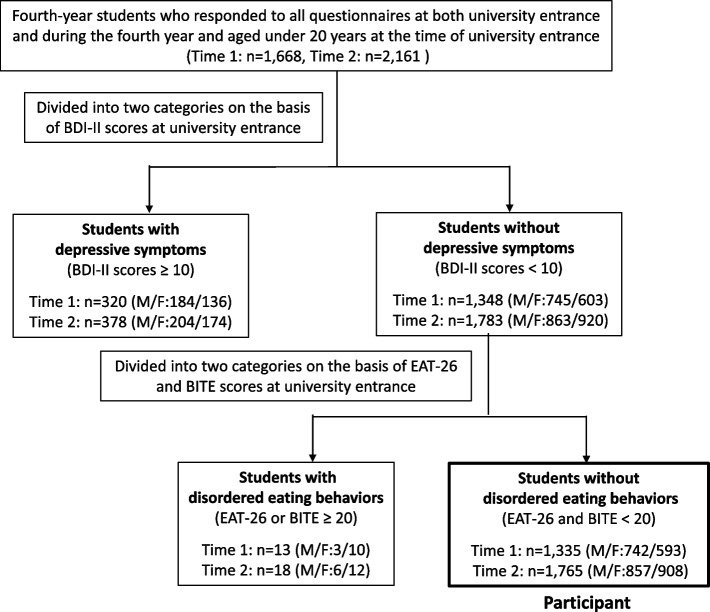
Participant flow chart. Time 1: before the pandemic, Time 2: during the pandemic. *Abbreviations* BDI-II, Beck Depression Inventory II, EAT-26, Eating Attitudes Test-26, BITE, Bulimic Inventory Test, Edinburgh

The data of 3,100 student, who did not have depressive symptoms at the clinical or subthreshold level, did not have disordered eating behavior at the time of university entrance, and completed questionnaires both at the time of university entrance and during their fourth year were available for analysis. Of these 1,335 were fourth-year students (742 male and 593 female) before the pandemic and 1,765 fourth-year students (857 male and 908 female) during the pandemic (Fig. 1). The annual checkups results are shown in Table 1.

**Table 1:** Results of an annual checkup

	Time 1		Time 2	
	Mean (SD)		Mean (SD)	
	First-year	Fourth-year	First-year	Fourth-year
Male	*n* = 742		*n* = 857	
Age (years)	18.2 (0.4)	21.2 (0.4)	18.2 (0.4)	21.2 (0.4)
BMI (kg/m^2^)	21.3 (3.0)	21.4 (3.0)	21.4 (3.1)	21.3 (3.0)
Female	*n* = 593		*n* = 908	
Age (years)	18.1 (0.3)	21.1 (0.3)	18.1(0.3)	21.1(0.3)
BMI (kg/m^2^)	20.3 (2.4)	20.5 (2.4)	20.4 (2.3)	20.4 (2.3)

Time 1: before the pandemic, Time 2: during the pandemic

*Abbreviations*: *SD* Standard deviation, *BMI* Body mass index

### Two-way repeated ANOVA results

Questionnaire results are shown in Table 2. For the male students, the two-way repeated ANOVA for the grade and time for each scale revealed significant interaction effects [BDI-II: *F*_(1,1597)_ = 12.56, *p* < 0.001; BITE: *F*_(__1,1597)_= 28.78, *p* < 0.001]. Next, we examined the simple main effects of grade and time. Fourth-year students during the pandemic scored significantly lower on the BDI-II and BITE than did fourth-year students before the pandemic (*p* < 0.001). Both before and during the pandemic, fourth-year students scored significantly higher on the BDI-II than they did as first-year students (*p* < 0.001). During the pandemic, fourth-year students scored significantly lower on the BITE than they did as first-year students (*p* < 0.001).

**Table 2:** Results of two-way repeated ANOVA

	Time 1		Time 2		*F*	*p*
	Mean (SD)		Mean (SD)		(grade × time)	
	First-year	Fourth-year	First-year	Fourth-year		
Male	*n* = 742		*n* = 857			
BDI-II	3.4 (2.7)	5.8 (5.5)	3.2 (2.7)	4.6 (6.0)	12.56	< 0.001^***^
EAT-26	2.5 (2.9)	1.9 (3.0)	2.6 (3.1)	1.8 (3.0)	0.82	0.36
BITE	3.9 (2.9)	4.1 (3.5)	3.9 (3.1)	3.2 (2.9)	28.78	< 0.001^***^
CISS-T	55.7 (10.8)	55.4 (10.7)	57.7 (10.6)	57.2 (11.2)	0.10	0.74
CISS-E	39.6 (10.2)	38.1 (10.3)	40.7 (10.0)	38.5 (10.5)	1.24	0.26
CISS-A	39.9 (10.8)	41.6 (10.5)	42.4 (11.3)	44.3 (10.7)	0.15	0.69
Female	*n* = 593		*n* = 908			
BDI-II	3.1 (2.6)	6.5 (5.7)	3.1 (2.6)	4.8 (6.1)	29.74	< 0.001^***^
EAT-26	2.9 (3.5)	2.7 (4.2)	3.4 (3.8)	2.5 (4.2)	7.33	0.007^**^
BITE	4.6 (3.6)	5.2 (4.9)	4.9 (3.7)	4.6 (4.6)	13.52	< 0.001^***^
CISS-T	54.8 (9.9)	53.2 (10.4)	55.2 (10.1)	54.7 (10.1)	4.36	0.03^*^
CISS-E	38.7 (9.5)	38.7 (9.6)	38.9 (9.3)	38.1 (10.1)	1.93	0.16
CISS-A	42.1 (9.9)	44.7 (9.1)	43.4 (10.5)	46.9 (9.8)	2.75	0.09

Time 1: before the pandemic, Time 2: during the pandemic

*Abbreviations: SD* standard deviation, *BDI-II* Beck Depression Inventory-II, *EAT-26* Eating Attitudes Test-26, *BITE* Bulimic Inventory Test, Edinburgh, *CISS* Coping inventory for stressful situations

^*^p < 0.05, ^**^p < 0.01, ^***^p < 0.001

For female students, the two-way repeated ANOVA revealed significant interaction effects [BDI-II: *F*_(__1,1499__)_ = 29.74, *p* < 0.001; EAT-26: *F*_(__1,1499__)_ = 7.33, *p* < 0.01; BITE:* F*_(__1,1499__)_ = 13.52, *p* < 0.001; CISS-T: *F*_(__1,1499__)_ = 4.36, *p* < 0.05]. We examined the simple main effects of grade and time. Fourth-year students during the pandemic scored significantly lower on the BDI-II (*p* < 0.001) and BITE (*p* < 0.01) and higher on the CISS-T (*p* < 0.01) than did fourth-year students before the pandemic. Both before and during the pandemic, fourth-year students scored significantly higher on the BDI-II than they did as first-year students (*p* < 0.001). During the pandemic, fourth-year students scored significantly lower on the EAT-26 than they did as first-year students (*p* < 0.001). Before the pandemic, fourth-year students scored significantly higher on the BITE (*p* < 0.01) and lower on the CISS-T (*p* < 0.001) than they did as first-year students.

### The depression status of fourth-year students

The results grouped by the depression status of the fourth year students are shown in Table 3. The chi-square test revealed significant differences before and during the pandemic (χ^2^(2) = 23.17, *p* < 0.001 for male and χ^2^(2) = 19.56, *p* < 0.001 for female students). The residual analysis revealed that the number in the nondepressed group was significantly greater during the pandemic (*n* = 733, 85.5%, asr = 3.4 for male and *n* = 760, 83.7%, asr = 4.4 for female students), and the number in the subthreshold group was significantly lower during the pandemic (*n* = 82, 9.6%, asr = -4.7 for male and *n* = 114, 12.6%, asr = -3.7 for female students).

**Table 3:** Depression status of fourth-year students

		Time 1	Time 2
Male			
Depression status			
Clinical	*n* (%)	25 (3.4)	42 (4.9)
	asr	-1.5	1.5
Subthreshold	*n* (%)	130 (17.5)	82 (9.6)
	asr	4.7	-4.7
Nondepressed	*n* (%)	587 (79.1)	733 (85.5)
	asr	-3.4	3.4
Female			
Depression status			
Clinical	*n* (%)	36 (6.1)	34 (3.7)
	asr	2.1	-2.1
Subthreshold	*n* (%)	116 (19.6)	114 (12.6)
	asr	3.7	-3.7
Nondepressed	*n* (%)	441 (74.3)	760 (83.7)
	asr	-4.4	4.4

Time 1: before the pandemic, Time 2: during the pandemic

*Abbreviation: asr* Adjusted standardized residual

### Comparison of CISS scores during the pandemic

For male students, the two-way repeated ANOVA on grade and group for the CISS showed significant interaction effects [CISS-T: *F*_(2,854)_ = 10.20, *p* < 0.001; CISS-E:* F*_(2,854)_ = 12.54, *p* < 0.001]. The results are shown in Table 4. Next, we examined the simple main effects of grade and group, and the results significantly differed. For the CISS-T scores, significant differences were observed between the nondepressed group and the clinical and subthreshold groups of fourth-year students (*p* < 0.001). The CISS-T scores of the clinical (*p* < 0.001) and subthreshold groups (*p* < 0.05) were significantly lower in the fourth-year than in the first-year. For the CISS-E scores, significant differences were observed between the nondepressed group and the clinical and subthreshold groups in both the fourth- (*p* < 0.001) and first-year (*p* < 0.05). A significant difference was also observed between the clinical group and the subthreshold group in the fourth-year (*p* < 0.01). The CISS-E scores of the clinical group was significantly increased (*p* < 0.01) and the score of the nondepressed group was significantly decreased (*p* < 0.001) in the fourth-year when compared to the first-year.

**Table 4:** Comparison of CISS scores during the pandemic

	Clinical		Subthreshold		Nondepressed		*F*	*p*
	Mean (SD)		Mean (SD)		Mean (SD)		(grade × group)	
	First-year	Fourth-year	First-year	Fourth-year	First-year	Fourth-year		
Male	*n* = 42		*n* = 82		*n* = 733			
CISS-T	57.8 (10.7)	50.1 (11.8)	55.6 (9.0)	52.9 (11.1)	58.0 (10.7)	58.1 (10.9)	10.20	< 0.001^***^
CISS-E	43.5 (9.9)	49.0 (9.2)	44.0 (10.1)	43.9 (9.8)	40.2 (9.9)	37.3 (10.1)	12.54	< 0.001^***^
CISS-A	36.7 (9.2)	40.8 (10.0)	42.7 (11.4)	43.0 (10.1)	42.7 (11.4)	44.7 (10.8)	1.56	0.21
Female	*n* = 34		*n* = 114		*n* = 760			
CISS-T	54.4 (11.3)	48.7 (11.2)	53.9 (10.1)	50.6 (8.9)	55.4 (10.1)	55.6 (10.0)	10.65	< 0.001^***^
CISS-E	43.7 (9.9)	50.8 (7.8)	40.3 (8.2)	44.0 (7.3)	38.4 (9.4)	36.6 (9.8)	24.76	< 0.001^***^
CISS-A	44.7 (11.7)	43.6 (8.8)	40.7 (10.0)	44.2 (8.1)	43.7 (10.4)	47.5 (9.9)	3.97	0.01^*^

*Abbreviation: SD* standard deviation, *CISS* Coping inventory for stressful situations

^*^p < 0.05, ^***^p < 0.001

For female students, the two-way repeated ANOVA on grade and group for the CISS scores showed significant interaction effects [CISS-T: *F*_(2,905)_ = 10.65, *p* < 0.001; CISS-E:* F*_(2,905)_ = 24.76, *p* < 0.001; CISS-A:* F*_(2,905)_ = 3.97, *p* < 0.05]. The results of the simple main effects of grade and group differed significantly. There were significant differences in the CISS-T (*p* < 0.001), CISS-E (*p* < 0.001) and CISS-A (*p* < 0.05) scores of the nondepressed group and the clinical and subthreshold groups in the fourth-year. For the CISS-T, the scores of the clinical and subthreshold groups were significantly lower in the fourth-year than in the first-year (*p* < 0.001). A significant difference was also observed between the clinical group and the nondepressed group in the first-year (*p* < 0.01). For the CISS-E, the scores of the clinical and subthreshold groups were significantly increased and the scores of the nondepressed group significantly decreased in the fourth-year as compared to the first-year (*p* < 0.001). There were significant differences between the clinical group and the subthreshold group in the fourth-year (*p* < 0.001), and between the clinical group and the nondepressed group in the first-year (*p* < 0.05). For the CISS-A, the scores of the nondepressed and subthreshold groups were significantly higher in the fourth-year than in the first-year (*p* < 0.001). A significant difference was also observed between the subthreshold group and the nondepressed group in the first-year (*p* < 0.05).

## Discussion

In this study, we investigated the effects of the pandemic on the depressive status and eating behaviors of fourth-year university students who did not have depressive symptoms or disordered eating behavior at the time of university entrance and examined whether changes in stress coping behavior are related to the development of depressive symptoms. We found that fourth-year students during the pandemic had a lower percentage of depressive symptoms and fewer bulimic symptoms. Female fourth-year students during the pandemic scored significantly higher on task-oriented stress coping ability than did students in their fourth-year before the pandemic. Additionally, a change in stress coping behavior was related to the development of depressive symptoms during the pandemic.

The BDI-II scores were significantly lower for fourth-year students during the pandemic than for fourth-year students before the pandemic, for both male and female students. There was a significant interaction effect between grade and time on BDI-II scores. Fourth-year students scored significantly higher on the BDI-II than they did when they entered university both before and during the pandemic. Our results showed that fourth-year students were more depressed than when they entered university. The fourth year is the final grade for most students, and the graduation period is a time of transition from student life to social life. There are practical issues such as deciding on a career path and job hunting, and many students tend to feel anxious about their career path and employment after graduation. However, the number of fourth-year students in the nondepressed group was significantly greater for students during the pandemic than for those before the pandemic, for both male and female students. The depressive symptoms of the fourth-year students attending during the pandemic did not worsen. There are several potential reasons for this. The cancelation of in-person classes and activities possibly provided students with more leisure time to engage in relaxing or creative endeavors, along with a reduced workload and less pressure to perform well academically [42]. Especially for fourth-year students, job hunts were primarily online during the pandemic in Japan, and many students felt that the burdens of academic equirements, transportation costs, and travel time had decreased. University students became accustomed to this new life, which may have helped them address academic and social stresses. Similar to these speculations, a study of Canadian teens revealed that although 40–46% reported feeling more depressed and anxious during the early phase of the pandemic, 20% reported feeling less depressed and 14% less anxious than before the pandemic [43]. A study of adolescents in China reported increased time available to spend on recreational and personal activities [42]. Increased free time and reduced pressure have emerged as key factors in improved emotional health during the pandemic [17]. Moreover, changes in stress coping behavior were related to the depressive status of the students in this study who attended during the pandemic. One possible interpretation of our results is that pandemic conditions may have had a positive effect on the depression status of fourth-year students, such as through more appropriate stress coping and from increased free time to attend to their mental health.

There was a significant interaction between the effects of grade and time on the EAT-26 and BITE scores of female students and on the BITE score of male students. Fourth-year students during the pandemic scored significantly lower on the BITE than did fourth-year students before the pandemic, both male and female students. We previously reported that depressive symptoms were correlated with the severity of the disordered eating behaviors of university students [43]. Depressive symptoms are a risk factor for EDs and are specifically associated with worsening bulimic symptoms [44]. Moreover, previous studies reported that difficulties in interpersonal relationships were potential factors in the development and maintenance of body image disturbances and altered eating behaviors [45, 46]. During the pandemic, students had fewer opportunities for interpersonal communication than before the pandemic, and social distancing may have provided a welcome respite [47]. Students may have experienced less difficulty in interpersonal relationships during the pandemic. Furthermore, they may have benefited from spending more time engaging in healthy eating routines [17]. In this study, one possible interpretation of the results is that a decrease in depressive symptoms and difficulty in interpersonal relationships may be associated with a decrease in disordered eating behaviors. However, further research is needed to address this issue.

Regarding stress coping, there was a significant interaction effect between grade and time on CISS-T scores, and female students in their fourth-year during the pandemic scored significantly higher on the CISS-T than did female students in their fourth-year before the pandemic. Additionally, there was a significant interaction effect between grade and group of depression on the CISS-T and CISS-E scores of both male and female students during the pandemic. During the pandemic, fourth-year students in the nondepressed group scored significantly higher on the CISS-T and significantly lower on the CISS-E than did students in the groups with increased depression, and the CISS-T scores of the increased-depression groups significantly decreased. Task-oriented coping is considered an adaptive coping behavior. Previous research has shown that coping strategies focused on task solving allow respondents to actively reduce feelings of uncertainty and increase feelings of control over their health [48]. The CISS-E score of the increased-depression groups significantly increased, and the score of the nondepressed group female students in their fourth-year during the pandemic was significantly decreased. Emotional-oriented coping is considered to be a nonadaptive aspect of coping and is associated with psychological distress [49]. A study in US students reported that the use of a coping strategy was a protective factor during the pandemic [21]. Our results suggest that decreased task-oriented stress coping and increased emotion-oriented stress coping may be associated with an increased risk of depressive symptoms, at both the clinical and subthreshold levels. Moreover, there was a significant interaction effect of grade and group on the CISS-A score of female students. Avoidance-oriented coping involves considered activities and cognitive changes to avoid stressful situations. In stressful situations such as the pandemic, this result suggested that avoidance-oriented coping might be effctive in preventing depressive symptoms, especially for female students. Previous studies reported that the effect of coping strategies on mental health might differ by sex [20]. The perceived risk associated with the pandemic was greater for women than for men, and thus, women were more active in practicing prevention in response to the pandemic [20]. Further research to examine possible gender differences is needed to address this issue.

### Limitations

This study has several limitations that need to be addressed in future research. First, it considered only depression status, eating behavior, and stress coping as effects of the pandemic. Future studies should consider anxiety and symptoms of other mental illnesses. Second, stress response can be affected by an individual’s background, social support, and many other factors. Future studies should assess these issues. Third, the number of participants before the pandemic was small because fewer students at that time underwent annual checkups in their fourth year. Annual checkups are not compulsory, so fewer students before the pandemic people came to campus when they were applying for jobs. Finally, our results are limited to a single university, fourth-year students, and a limited time period. Research on the mental health of students should be expanded to other years and to include other universities in future studies.

## Conclusions

Our study revealed that fourth-year university students during the COVID-19 pandemic who did not have depressive symptoms at the time of university entrance had a lower percentage of depressive symptoms and fewer bulimic symptoms than did students enrolled before the pandemic. Additionally, the results suggest that the use of task-oriented coping may have been associated with a decreased risk of developing depressive symptoms during the pandemic, especially for female students.

## Data Availability

All data generated or analyzed during this study are included in this article. Further enquiries can be directed to the corresponding author.

## Abbreviations

COVID-19: Coronavirus disease 2019
EDs: Eating disorders
BDI-II: Beck Depression Inventory II
EAT-26: Eating Attitudes Test-26
BITE: Bulimic Inventory Test, Edinburgh
CISS: Coping Inventory for Stressful Situations
ANOVA: Analysis of variance

## References

[1] Yuan K, Zheng YB, Wang YJ, Sun YK, Gong YM, Huang YT, et al. A systematic review and meta-analysis on prevalence of and risk factors associated with depression, anxiety, and insomnia in infectious diseases, including COVID-19: A call to action. Mol Psychiatry. 2022;27:3214–22. 10.1038/s41380-022-01638-z. 35668158 10.1038/s41380-022-01638-z PMC9168354

[2] Favreau M, Hillert A, Osen B, Gärtner T, Hunatschek S, Riese M, et al. Psychological consequences and differential impact of the COVID-19 pandemic in patients with mental disorders. Psychiatry Res. 2021;302: 114045. 10.1016/j.psychres.2021.114045. 34126461 10.1016/j.psychres.2021.114045 PMC8180351

[3] Yamamoto T, Uchiumi C, Suzuki N, Sugaya N, Murillo-Rodriguez E, Machado S, et al. Mental health and social isolation under repeated mild lockdowns in Japan. Sci Rep. 2022;12:8452. 10.1038/s41598-022-12420-0. 35589930 10.1038/s41598-022-12420-0 PMC9118820

[4] Nagib N, Horita R, Miwa T, Adachi M, Tajirika S, Imamura N, et al. Impact of COVID-19 on the mental health of Japanese university students (years II-IV). Psychiatry Res. 2023;325:115244. 10.1016/j.psychres.2023.11524. 10.1016/j.psychres.2023.115244 37182282

[5] Son C, Hegde S, Smith A, Wang X, Sasangohar F. Effects of COVID-19 on college students’ mental health in the United States: Interview survey study. J Med Internet Res. 2020;22: e21279. 10.2196/21279. 32805704 10.2196/21279 PMC7473764

[6] Takagaki K, Yokoyama S. Factors associated with university students’ deterioration from subthreshold depression to depression before and during the COVID-19 pandemic. Behav Sci (Basel). 2023;13:72. 10.3390/bs13010072. 36661644 10.3390/bs13010072 PMC9854505

[7] Fruehwirth JC, Biswas S, Perreira KM. The Covid-19 pandemic and mental health of first-year college students: examining the effect of Covid-19 stressors using longitudinal data. PLoS ONE. 2021;16: e0247999. 10.1371/journal.pone.0247999. 33667243 10.1371/journal.pone.0247999 PMC7935268

[8] Li Y, Wang A, Wu Y, Han N, Huang H. Impact of the COVID-19 pandemic on the mental health of college students: a systematic review and meta analysis. Front Psychol. 2021;12: 669119. 10.3389/fpsyg.2021.669119. 34335381 10.3389/fpsyg.2021.669119 PMC8316976

[9] Wang C, Wen W, Zhang H, Ni J, Jiang J, Cheng Y, et al. Anxiety, depression, and stress prevalence among college students during the COVID-19 pandemic: a systematic review and meta-analysis. J Am Coll Health. 2023;71:2123–30. 10.1080/07448481.2021.1960849. 34469261 10.1080/07448481.2021.1960849

[10] Javadi-Pashaki N, Darvishpour A. Survey of stress and coping strategies to predict the general health of nursing staff. J Educ Health Promot. 2019;8:74. 10.4103/jehp.jehp_355_18. 31143791 10.4103/jehp.jehp_355_18 PMC6512229

[11] Kurisu K, Matsuoka M, Sato K, Hattori A, Yamanaka Y, Nohara N, et al. Increased prevalence of eating disorders in Japan since the start of the COVID-19 pandemic. Eat Weight Disord. 2022;27:2251–5. 10.1007/s40519-021-01339-6. 34855142 10.1007/s40519-021-01339-6 PMC8638639

[12] Devoe DJ, Han A, Anderson A, Katzman DK, Patten SB, Soumbasis A, et al. The impact of the COVID-19 pandemic on eating disorders: a systematic review. Int J Eat Disord. 2023;56:5–25. 10.1002/eat.23704. 35384016 10.1002/eat.23704 PMC9087369

[13] Kawai K, Tachimori H, Yamamoto Y, Nakatani Y, Iwasaki S, Sekiguchi A, et al. Trends in the effect of COVID-19 on consultations for persons with clinical and subclinical eating disorders. Biopsychosoc Med. 2023;17:29. 10.1186/s13030-023-00285-2. 37559073 10.1186/s13030-023-00285-2 PMC10410894

[14] Nagl M, Jacobi C, Paul M, Beesdo-Baum K, Höfler M, Lieb R, et al. Prevalence, incidence, and natural course of anorexia and bulimia nervosa among adolescents and young adults. Eur Child Adolesc Psychiatry. 2016;25:903–18. 10.1007/s00787-015-0808-z. 26754944 10.1007/s00787-015-0808-z

[15] Stice E, Marti CN, Rohde P. Prevalence, incidence, impairment, and course of the proposed DSM-5 eating disorder diagnosis in 8-year prospective community study of young women. J Abnorm Psychol. 2013;122:445–57. 10.1037/a0030679. 23148784 10.1037/a0030679 PMC3980846

[16] Shanahan L, Steinhoff A, Bechtiger L, Murray AL, Nivette A, Hepp U, et al. Emotional distress in young adults during the COVID-19 pandemic: evidence of risk and resilience from a longitudinal cohort study. Psychol Med. 2022;52:824–33. 10.1017/S003329172000241X. 32571438 10.1017/S003329172000241X PMC7338432

[17] Silk JS, Scott LN, Hutchinson EA, Lu C, Sequeira SL, McKone KMP, et al. Storm Clouds and Silver Linings: Day-to-Day Life in COVID-19 Lockdown and Emotional Health in Adolescent Girls. J Pediatr Psychol. 2022;47:37–48. 10.1093/jpepsy/jsab107. 34664665 10.1093/jpepsy/jsab107 PMC8574543

[18] Shiraishi N, Sakata M, Toyomoto R, Yoshida K, Luo Y, Nakagami Y, et al. Dynamics of depressive states among university students in Japan during the COVID-19 pandemic: an interrupted time series analysis. Ann Gen Psychiatry. 2023;22:38. 10.1186/s12991-023-00468-9. 37814328 10.1186/s12991-023-00468-9 PMC10563354

[19] Ihm L, Zhang H, van Vijfeijken A, Waugh MG. Impacts of the Covid-19 pandemic on the health of university students. Int J Health Plann Manage. 2021;36:618–27. 10.1002/hpm.3145. 33694192 10.1002/hpm.3145 PMC8206857

[20] Rana IA, Bhatti SS, Aslam AB, Jamshed A, Ahmad J, Shah AA. COVID-19 risk perception and coping mechanisms: Does gender make a difference? Int J Disaster Risk Reduct. 2021;55: 102096. 10.1016/j.ijdrr.2021.102096. 33777688 10.1016/j.ijdrr.2021.102096 PMC7987376

[21] Waselewski EA, Waselewski ME, Chang T. Needs and Coping Behaviors of Youth in the US During COVID-19. J Adolesc Health. 2020;67:649–52. 10.1016/j.jadohealth.2020.07.043. 32933836 10.1016/j.jadohealth.2020.07.043

[22] Bhagyalakshmi M, Ramana BV, Suresh H, Raj JM. Assessment of the level of stress and coping strategies among patients with coronary artery. J Sci Soc. 2012;39:136–40. 10.4103/0974-5009.105918.

[23] Mahmoud JS, Staten R, Hall LA, Lennie TA. The relationship among young adult college students' depression, anxiety, stress, demographics, life satisfaction, and coping styles. Issues Ment Health Nurs. 2012;33:149–56. 10.3109/01612840.2011.632708. 10.3109/01612840.2011.632708 22364426

[24] Ball K, Lee C. Psychological stress, coping, and symptoms of disordered eating in a community sample of young Australian women. Int J Eat Disord. 2002;31:71–81. 10.1002/eat.1113. 11835300 10.1002/eat.1113

[25] Manchia M, Gathier AW, Yapici-Eser H, Schmidt MV, de Quervain D, van Amelsvoort T, et al. The impact of the prolonged COVID-19 pandemic on stress resilience and mental health: A critical review across waves. Eur Neuropsychopharmacol. 2022;55:22–83. 10.1016/j.euroneuro.2021.10.864. 34818601 10.1016/j.euroneuro.2021.10.864 PMC8554139

[26] Ng QX, Chee KT, De Deyn ML, Chua Z. Staying connected during the COVID-19 pandemic. Int J Soc Psychiatry. 2020;66:519–20. 10.1177/0020764020926562. 32380875 10.1177/0020764020926562 PMC7405627

[27] Jinnin R, Okamoto Y, Takagaki K, Nishiyama Y, Yamamura T, Okamoto Y, et al. Affiliations expand et al. Detailed course of depressive symptoms and risk for developing depression in late adolescents with subthreshold depression: a cohort study. Neuropsychiatr Dis Treat. 2016;13:25–33. 10.2147/NDT.S117846. 10.2147/NDT.S117846 PMC5191576 28053534

[28] Beck A, Steer R, Brown G. Manual for the Beck Depression Inventory-II. 1996. San Antonio, TX: Psychological Corporation.

[29] Arnau RC, Meagher MW, Norris MP, Bramson R. Psychometric evaluation of the Beck Depression Inventory-II with primary care medical patients. Health Psychol. 2001;20:112–9. 10.1037/0278-6133.20.2.112. 11315728 10.1037//0278-6133.20.2.112

[30] Garner DM, Olmsted MP, Bohr Y, Garfinkel PE. The eating attitudes test: psychometric features and clinical correlates. Psychol Med. 1982;12:871–8. 10.1017/s0033291700049163. 6961471 10.1017/s0033291700049163

[31] Henderson M, Freeman CP. A self-rating scale for bulimia. The ‘BITE’. Br J Psychiatry. 1987;150:18–24. 10.1192/bjp.150.1.18. 10.1192/bjp.150.1.18 3651670

[32] Endler NS, Parker JDA. Coping inventory for stressful situations (CISS): manual. Toronto: Malti- Health Systems Inc; 1990.

[33] Kojima M, Furukawa TA, Takahashi H, Kawai M, Nagaya T, Tokudome S. Cross-cultural validation of the Beck Depression Inventory-II in Japan. Psychiatry Res. 2002;110:291–9. 10.1016/s0165-1781(02)00106-3. 12127479 10.1016/s0165-1781(02)00106-3

[34] Kojima M, Furukawa T. Japanese Version of the Beck Depression Inventory. (2nd ed). 2003. Nippon-Hyoron-sha Co, Tokyo.

[35] Mann AH, Wakeling A, Wood K, Monck E, Dobbs R, Szmukler G. Screening for abnormal eating attitudes and psychiatric morbidity in an unselected population of 15-year-old schoolgirls. Psychol Med. 1983;13:573–80. 10.1017/s0033291700047991. 6622610 10.1017/s0033291700047991

[36] Siervo M, Boschi V, Papa A, Bellini O, Falconi C. Application of the SCOFF, eating attitudes test 26 (EAT 26) and eating inventory (TFEQ) questionnaires in young women seeking diet-therapy. Eat Weight Disord. 2005;10:76–82. 10.1007/BF03327528. 16114220 10.1007/BF03327528

[37] Nakai Y. Validity of the Japanese version of eating attitudes test (EAT). Seishin Igaku. 2003;45:161–5. 10.11477/mf.1405100638.

[38] Mukai T, Crago M, Shisslak CM. Eating attitudes and weight preoccupation among female high school students in Japan. J Child Psychol Psychiatry. 1994;35:677–88. 10.1111/j.1469-7610.1994.tb01213.x. 8040220 10.1111/j.1469-7610.1994.tb01213.x

[39] Nakai Y, Noma S. Evaluation method of eating disorder symptoms. Modern Physician. 2007;27:785–8.

[40] Furukawa T, Suzuki-Moor A, Saito Y, Hamanaka T. Reliability and validity of the Japanese version of the coping inventory for stressful situations (CISS): a contribution to the cross-cultural studies of coping. Seishin Shinkeigaku Zasshi. 1993;95:602–20. 10.2466/08.02.PR0.116k23w6. 8234537

[41] Watanabe K, Yokoyama K, Furukawa TA. Reliability and validity of the Japanese version of the coping inventory for adults for stressful situations in healthy people. Psychol Rep. 2015;116:447–69. 10.2466/08.02.PR0.116k23w6. 25826435 10.2466/08.02.PR0.116k23w6

[42] Tang S, Xiang M, Cheung T, Xiang Y-T. Mental health and its correlates among children and adolescents during COVID-19 school closure: The importance of parent-child discussion. J Affect Disord. 2021;279:353–60. 10.1016/j.jad.2020.10.016. 33099049 10.1016/j.jad.2020.10.016 PMC7550131

[43] Miyake Y, Okamoto Y, Takagaki K, Yoshihara M. Changes in Eating Attitudes and Risk for Developing Disordered Eating Behaviors in College Students with Subthreshold Eating Disorders: A Cohort Study. Psychopathology. 2023;56:276–84. 10.1159/000527604. 10.1159/000527604 36509080

[44] Okamoto Y, Miyake Y, Nagasawa I, Yoshihara M. Cohort survey of college students’ eating attitudes: interventions for depressive symptoms and stress coping were key factors for preventing bulimia in a subthreshold group. BioPsychoSoc Med. 2018;12:8. 10.1186/s13030-018-0127-y. 29849751 10.1186/s13030-018-0127-y PMC5968577

[45] Fairburn CG, Cooper Z, Shafram R. Cognitive behaviour therapy for eating disorders: a transdiagnostic theory and treatment. Behav Res Ther. 2003;41:509–28. 10.1016/s0005-7967(02)00088-8. 12711261 10.1016/s0005-7967(02)00088-8

[46] Miyake Y, Okamoto Y, Onoda K, Shirao N, Okamoto Y, Yamawaki S. Brain activation during the perception of stressful word stimuli concerning interpersonal relationships in anorexia nervosa patients with high degrees of alexithymia in an fMRI paradigm. Psychiatry Res. 2012;201:113–9. 10.1016/j.pscychresns.2011.07.014. 22398299 10.1016/j.pscychresns.2011.07.014

[47] Hawes MT, Szenczy AK, Klein DN, Hajcak G, Nelson BD. Increases in depression and anxiety symptoms in adolescents and young adults during the COVID-19 pandemic. Psychol Med. 2022;52:3222–30. 10.1017/S0033291720005358. 10.1017/S0033291720005358 PMC7844180 33436120

[48] Savary A, Hammouda M, Genet L, Godet C, Bunel V, Weisenburger G, et al. Coping strategies, anxiety and depression related to the COVID-19 pandemic in lung transplant candidates and recipients. Results from a monocenter series. Respir Med Res. 2021; 80:100847. 10.1016/j.resmer.2021.100847. 10.1016/j.resmer.2021.100847 PMC8260501 34371237

[49] Spoor ST, Bekker MH, van Strien T, van Heck GL. Relations between negative affect, coping, and emotional eating. Appetite. 2007;48:368–76. 10.1016/j.appet.2006.10.005. 17145096 10.1016/j.appet.2006.10.005

